# Imbalanced positive selection maintains the functional divergence of duplicated *DIHYDROKAEMPFEROL 4-REDUCTASE* genes

**DOI:** 10.1038/srep39031

**Published:** 2016-12-14

**Authors:** Bing-Hong Huang, Yi-Wen Chen, Chia-Lung Huang, Jian Gao, Pei-Chun Liao

**Affiliations:** 1 Department of Life Science, National Taiwan Normal University, Taipei 11677, Taiwan; 2 Department of Biological Science and Technology, National Pingtung University of Science and Technology, Pingtung 91201, Taiwan; 3 The Key Laboratory for Silviculture and Conservation of Ministry of Education, College of Forestry, Beijing Forestry University, Beijing 100083, China

## Abstract

Gene duplication could be beneficial by functional division but might increase the risk of genetic load. The dynamics of duplicated paralogs number could involve recombination, positive selection, and functional divergence. Duplication of *DIHYDROFLAVONOL 4-REDUCTASE* (*DFR*) has been reported in several organisms and may have been retained by escape from adaptive conflict (EAC). In this study, we screened the angiosperm *DFR* gene focusing on a diversified genus *Scutellaria* to investigate how these duplicated genes are retained. We deduced that gene duplication involved multiple independent events in angiosperms, but the duplication of *DFR* was before the divergence of *Scutellaria*. Asymmetric positive selective pressures resulted in different evolutionary rates between the duplicates. Different numbers of regulatory elements, differential codon usages, radical amino acid changes, and differential gene expressions provide evidences of functional divergence between the two *DFR* duplicates in *Scutellaria*, implying adaptive subfunctionalization between duplicates. The discovery of pseudogenes accompanying a reduced replacement rate in one *DFR* paralogous gene suggested possibly leading to “loss of function” due to dosage imbalance after the transient adaptive subfunctionalization in the early stage of duplication. Notwithstanding, episodic gene duplication and functional divergence may be relevant to the diversification of ecological function of *DFR* gene in *Scutellaria*.

Duplication of plant metabolic genes is not uncommon^1^. The variability and plasticity of ancestral secondary metabolism genes enabled the plants to adapt to environmental changes^1^,^2^, with the selective forces conceivably changing repeatedly owing to the continuously changing environment^1^. Recombination and positive selection comprise the two main factors preserving and accelerating genetic variations of “old genes”. Gene duplication affords not only increased tolerance of harmful or detrimental mutations but also opportunities to create new functions. The maintenance of gene copies of floral characters is usually justified as neofunctionalization (neoF) and subfunctionalization (subF) driven by positive selection^3^. Although balancing selection was suggested as one of the mechanisms for the maintenance of divergent (neoF) or complementary (subF) functions of functional gene copies^4^,^5^, several studies indicated that positive divergent selection and duplication events act as reciprocal evolutionary forces driving adaptive trait diversification^6^,^7^. Flagel and Wendel^8^ suggested that unequal crossing-over and/or gene conversion would homogenize duplicates, providing a means of amplifying adaptively important genes, with a tendency to accelerate the divergence of non-recombining clusters, and permitted gene family diversification and evolutionary plasticity cf. plant resistance genes^9^,^10^. The functional divergence of duplicates could be retained by positive selection while recombination ensured the pleiotropic effect^11^. However, duplicated genes usually have only minor sequence variations that are sufficient to alter the substrate and product specificity but, thus, possess insufficient characteristics to predict their functional divergence^1^. On the other hand, both concerted evolution and purifying selection retained only small variations between paralogous genes for a long time, ensuring that the functional constraints of duplicated genes paralleled their expression in different tissues (i.e., subF)^6^,^7^.

Anthocyanins have been proposed to function in plant adaptation and interactions with animals, e.g., attracting pollinators and frugivores, and/or repelling herbivores and parasites^12^. Accelerated evolutionary rates of downstream genes of the anthocyanin biosynthetic pathway (ABP)^13^,^14^,^15^ suggested that the ecological functions of anthocyanins were mostly contributed by the rapid evolution of these genes. One ABP protein, a dihydroflavonol 4-reductase (DFR), is located in a metabolic node that exhibits a strong stereo-specificity and varies with respect to the acceptance of dihydroflavonol substrates with different B-ring oxidation states, which is thought to engineer the flower color in different plant species^16^,^17^. In addition, because DFR diverts the conversion of precursor flavonoids into anthocyanins, proanthocyanidins, and phlobaphenes^18^, it was suggested to play pleiotropic roles in plant resistance to pathogen infections, starch level regulation, etc.^19^. Most ABP genes, including *DFR*, are single-copy genes in several angiosperm species (e.g., *Arabidopsis*, *Oryza*, *Vitis*). However, certain studies revealed *DFR* gene duplication in other species^20^,^21^,^22^,^23^,^24^ and multiple copies of ABP genes were often found to be linked to whole genome duplication or tandem duplication (e.g., *Brassica rapa*^25^, *Lotus japonicus*^26^, *Ipomoea* sp.^27^). These duplicates may be differentially expressed in different tissues by using varied promoters^28^. Sequence analyses and enzymatic assays provided evidence for an “escape from adaptive conflict” (EAC) evolutionary model of subF for the duplicates of *DFR*^29^. Because of such physiologically and ecologically important functions, similarly to other downstream ABP genes, *DFR* was assumed to be adaptively divergent, between the duplicates and between ecologically divergent taxa.

Transcriptomic analyses of inflorescence buds of *Scutellaria* (Labiatae) have indicated that transcription factors *R2R3-MYBs* that regulate the expression of ABP genes underwent recent duplication events and were positively selected for functional divergence^30^. Like many studies concerning the translational level adaptive divergence of downstream ABP genes [e.g., *ANCYOCYANIDIN SYNTHASE* (*ANS*) and *UDP-GLUCOSE: FLAVONOID 3-OXY-GLUCOSYLTRANSFERASE* (*UFGT*)]^13^,^14^,^15^, such transcriptional level adaptive divergence of transcription factors was suggested to be related to a rapid speciation of phylogenetically related *Scutellaria* species in Taiwan^30^. Taiwanese *Scutellaria* have originated at least three times and the time of divergence could be traced back to ~0.61 Mya, with local speciation events between 0.2 Mya and 0.02 Mya^31^. The ragged topography of Taiwan Island warranties habitat heterogeneity and imposes geographical barriers increasing the reproductive isolation between Taiwanese *Scutellaria* species^31^. Heterogeneous environments create the opportunity for adaptive divergence among phylogenetically close lineages^32^,^33^. Since the floral colors are usually associated with adaptive traits that affect the fitness and undergo selection^34^,^35^, duplications of color-related genes became relevant for the enhancement of trait divergence (e.g., changing the pollination syndrome) and for the acceleration of the speciation rate^34^,^36^. Similarly to many other plants, the duplication of *DFR* was also found in *Scutellaria* which has diversified floral colors among various species^30^. Therefore, this constitutes a unique opportunity to test whether this gene duplication was adaptively annotated and what kind of evolutionary pressures led the coexistence of these duplicated paralogous genes in the genome.

Here, we asked two questions: (1) Is the common phenomenon of *DFR* duplication in angiosperms a relic of ancient whole genome duplications, or alternatively, a consequence of multiple duplication episodes in several organisms? (2) In a group of diversified species of *Scutellaria*, does a selective pressure exist on *DFR* and what kind of evolutionary mechanism lead the duplicated paralogs to persist in the genome? To answer these questions, phylogenetic and population genetics analyses of hundreds of angiosperm *DFR* sequences were conducted to address the sequence of duplication and speciation events. Based on the *DFR* sequences of *Scutellaria*, we focused on the recombination and positive selection to explain the persistence of paralogous duplicates. Gene expression analysis was also used for confirming the subF of paralogs of *DFR* in *Scutellaria*. Herein, we present an unusual evolutionary fate of this ecologically important gene and suggest that gene duplication may be implicated in the diversity and adaptation of anthocyanin pathway genes.

## Results and Discussion

### Multiple independent duplication events of angiosperm *DFR* genes

In the collected sequence data, we identified *DFR* duplication in several genera, including *Aegilops, Allium, Brassica, Chrysanthemum, Convolvulus, Cyclamen, Epimedium, Glycine, Ipomoea, Lotus, Medicago, Nicotiana, Petunia, Pyrus, Scutellaria, Triticum, Turbina, Vaccinium*, etc. Two competing hypotheses were proposed to explain the widespread duplication events: (1) ancient genome duplication with an ensuing loss of duplicates in certain taxa and (2) multiple independent duplication events among various taxa of angiosperms. If the first hypothesis was true, we expected a single cluster of each orthologous *DFR* from different taxa in the phylogeny; in contrast, if independent duplication was the case, multiple clusters associated with taxonomic groups were expected. Therefore, we constructed a gene tree using 407 coding sequences of *DFR* and found that duplication clusters were widespread in the angiosperm phylogeny (Supplementary Fig. 1), supporting multiple independent duplication events.

Several whole genome duplication events took place during early angiosperm evolution that led to shared synteny between two or more sets of chromosomes^37^. However, many ABP genes, including *DFR*, remain in a single or low copy state, resulting from a very recent duplication. In other words, all *DFR* copies derived from the ancient whole genome duplication event were lost during angiosperm diversification. *DFR* are pleiotropic genes responsible for flavonoid precursor metabolism and Paterson *et al*.^38^ suggested that structural or metabolic genes were preferentially fractionated (loss or reversal to single copy state) after whole genome duplication. Similarly, Li *et al*.^39^ indicated that genes with high connectivity in the regulation networks or pleiotropic genes were also preferentially fractionated. These observations supported the hypothesis that the expansion of *DFR* copy number through ancient polyploidy is less possible than independent duplications among various species of angiosperms.

### Preservation of ancestral polymorphisms by recombination of angiosperm *DFR*

**Figure 1:**
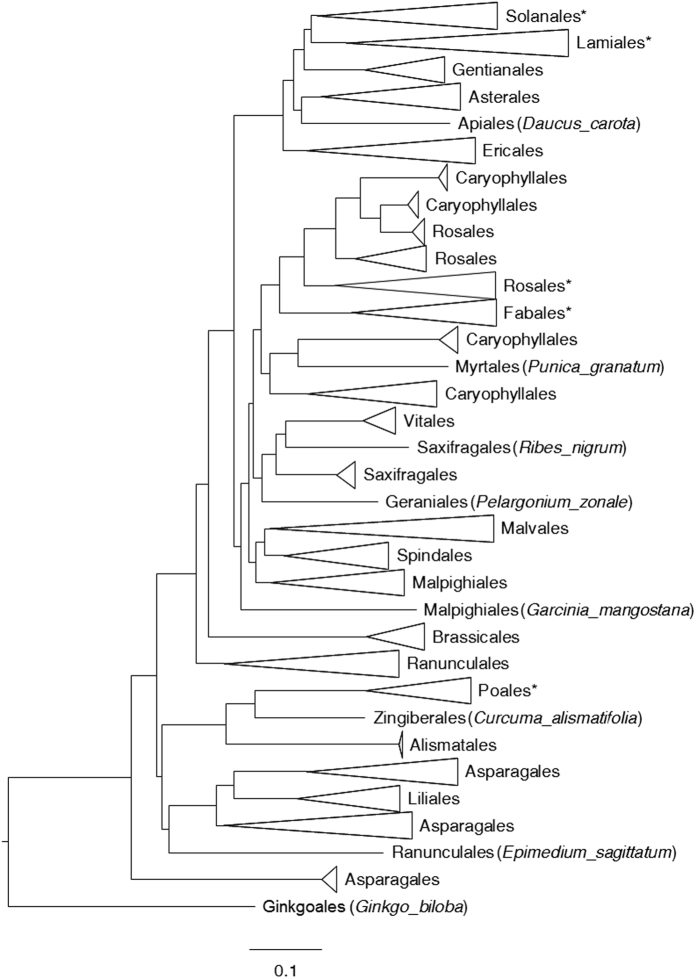
The neighbor-joining tree of *DFR* gene. Detailed evolutionary relationships of the lineages are shown in Supplementary Fig. 1. Lineages with potential duplications or duplications identified previously have been labeled with star (*).

However, the phylogenetic topology of *DFR* gene in Fig. 1 was slightly inconsistent with the species tree suggested by APG IV^40^. A mutation that associates with the classes in which it arose will eventually “migrate” to different alleles in the course of recombination^41^,^42^, explaining the inconsistency between *DFR* gene tree and APG IV classification, and the recombination is suggested as an important mechanism in maintaining ancestral polymorphism. The recombination rate *R* estimated from 406 angiosperm *DFR* sequences was 0.1322 between adjacent sites, with minimum recombination events (*Rm*) 37 times, and the *ZZ* statistic of 0.0334. These estimates suggested that the variation of *DFR* might have been more or less driven by recombination at the early stages of angiosperm diversification. We proposed that if recombination facilitates the preservation of ancestral polymorphisms among the taxa, the nucleotide variation estimated by pairwise differences would be larger than that estimated by the number of segregations (i.e., Tajima’s *D* > 0), i.e., similarly to the consequence of balancing selection. Although Tajima’s *D* was usually used for population-level studies, we used it to compare the amount of nucleotide differences accumulated between the past and the recent past in different taxa. A significant positive *D* of the angiosperms (*D* = 3.74047, *P* < 0.001) indicated accumulation of ancient polymorphisms exceeding that of newly derived variations. The high values of *R* and moderate *Rm* and *ZZ*, taken together with the positive Tajima’s *D*, suggested that the current genetic diversity of *DFR* was predominantly derived from the ancestral polymorphism after angiosperm diversification. Such recombinant duplicates of *DFR* have been already evidenced by *in vitro* experiments to be functionally divergent and expressed in different tissues at different developmental stages^17^.

Recombination is a common mechanism driving and maintaining the genetic diversity, and providing variations (agents) for selection, but at the same time it can also comprise a trade-off to increase genetic loads^43^,^44^. Therefore, we hypothesized that the signals of the balancing selection would not be found throughout the evolutionary trajectory of angiosperms but only in certain lineages. Hence, we further tested the recombination rate of each genus (rather than all angiosperms) to search for evidence of balancing selection throughout the evolutionary history of angiosperms. Coalescent *Rm* simulations for the lineages within genera revealed observed values that were non-significantly greater than the expected values (Table 1), suggesting that the evolution of *DFR* was to a less degree affected by historical recombination at the generic level. Non-deviation from neutral evolution inferred by Tajima’s *D* test also suggested that the accumulation of common nucleotide polymorphisms was too small to contribute to *DFR* variation at the genetic level. However, a significantly higher than expected intragenic recombination estimated by coalescent *ZZ* statistic in genera *Scutellaria*, *Ipomoea*, and *Triticum*, indicated that the increased recombination nonetheless reshuffled the nucleotide variation in certain specific taxa in the recent past (Table 1).

Taken together, ancient recombination preserved ancestral polymorphisms of *DFR* in the angiosperms but was less frequent in most taxa in the recent past. The evolutionary pressure of the balancing selection can thus only act on specific lineages of angiosperms instead of being a general phenomenon, implying the existence of some other evolutionary forces, in addition to the balancing selection, driving the current diversity of *DFR* at generic or species level.

### Duplication and positive selection dominate the evolution of *DFR* in *Scutellaria*

**Figure 2:**
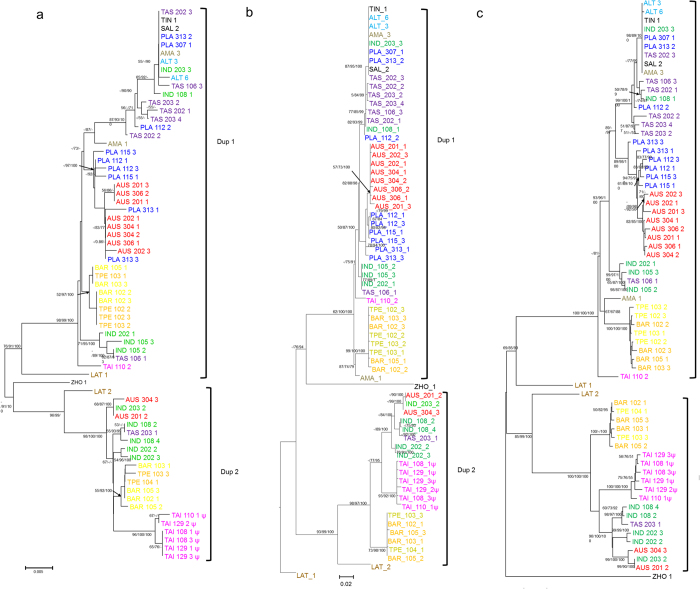
The neighbor-joining trees of *DFR* gene in *Scutellaria* constructed with exon (**a**) and intron (**b**) nucleotide sequences, and amino acid sequences (**c**). Branch support value (including bootstrap of NJ and ML, and posterior probability of BI, respectively) > 50% are shown adjacent to the nodes. Sequences of different species are indicated by different colors. Species with no identified duplicated *DFR* are indicated in black. Dup1 and Dup2 denote two putative duplications.

Duplication events involving *DFR* have been reported in several species, e.g., *Zea mays* and *Teosinte guerrero*^20^, *Ipomoea nil* and *I. purpurea*^21^, *Medicago truncatula*^22^, *Lotus japonicus*^23^, *Populus trichocarpa*^24^, etc. In *Scutellaria*, gene duplication has been also evidenced by distinguishable intron lengths (Supplementary Fig. 2) and distinguishable clusters in phylogenetic analyses (Dup1 and Dup2, Fig. 2). All of the phylogenetic analysis placed one copy from each species in a separate clade, indicating an ancient duplication before *Scutellaria* divergence that resulted in two paralogs. Topological tests showed that the evolutionary scenario “duplication after speciation” was rejected by Approximately Unbiased (AU), Kishino–Hasegawa (KH), and Shimodaira–Hasegawa (SH) tests (*P* = 0.006, 0.007, and 0.007, respectively), suggesting that the duplication event has occurred before *Scutellaria* species divergence (Supplementary Table 2).

Phylogenetic analyses revealed that certain lineages were misassigned to a different clade in the exon tree or in the amino acid tree, which was probably caused by the long-branch attraction or positive selection, e.g., LAT1 (*S. lateriflora*) and ZHO1 (*S. zhongdianensis*) (Fig. 2). Furthermore, high heterozygote frequency was observed in several *Scutellaria* species samples in both duplicates, and such high heterozygosity could be a result of balancing selection. Therefore, we re-estimated Tajima’s *D* and the recombination rate of *Scutellaria DFR* using full-length sequences (exons+introns) to test whether the misidentification and diversification were associated with the balancing selection. Here, we anticipated a positive Tajima’s *D* and a higher recombination rate in exons than in introns, should the balancing selection lead to high *DFR* polymorphism. However, non-deviation from zero of Tajima’s *D* suggested a failure to reject the neutral model (*D* = 0) and did not support the hypothesis of the balancing selection. In addition, the recombination rate of full-length sequences was 0.0034 for adjacent sites, similar to exon only estimates (*R* = 0.0038, Table 1), indicating that the recombination did not occur only at exons. This implied that the balancing selection could be not the driving force responsible for the diversification of *DFR* in *Scutellaria*. On the other hand, high ω value (346.74) was found in LAT1 when using the exon tree as the input tree in the free-ratio model, which fit the observations better than the constant model (M0; LRT: 2Δ*L* = 107.168, *P* = 0.007, Supplementary Table 3). On the other hand, LAT1 had low ω value (0.3640) when using the intron tree as the input tree in the free-ratio model, suggesting that positive selective pressures might affect the tree topology of *DFR* genes. In addition to LAT1, there were 42 branches with ω > 1 in the exon tree but only 21 branches with ω > 1 in the intron tree, suggesting that the positive selection was dominant in the evolution of *Scutellaria DFR* (Supplementary Fig. 3). In fact, the branches with ω > 1 mostly had an estimated dS = 0, indicating a selectively advantage of accumulation of amino acid mutations in *Scutellaria* DFR and also suggesting episodic diversifying selection dominating *DFR* variation e.g.^45^).

We also found higher frequency of branches with ω > 1 in clade Dup1 [15/88 (17.0%) in the intron tree; 33/89 (37.1%) in the exon tree] than in Dup2 [6/41 (14.6%) in the intron tree; 9/45 (20.0%) in the exon tree] with the free-ratio model (Supplementary Fig. 3), implying different evolutionary fates between the two *DFR* duplicates. The branch-site-model test indicated ω > 1 estimates for all lineages of the Dup1 clade (LRT: 2Δ*L* = 18.066, *P* = 1.121×10^−5^, Table 2), suggesting that positive selection drove the diversification of Dup1. However, the lineage of the entire Dup1 clade failed to reject the null model (foreground ω = 1 fixed, LRT: 2Δ*L* = 0.476, *P* = 0.456). Similar inference of ω > 1 was obtained for all lineages of the Dup2 clade but not for the branch of the whole Dup2 clade (Table 2). These results suggested that the signatures of positive selection were not detected after gene duplication but, instead, following species divergence, which means that the diversification of each single *DFR* paralog was advantageous to species adaptation. However, it is worth noting that the greatest divergence between Dup1 and Dup2 was contributed by synonymous mutations (synonymous nucleotide divergence 0.169 vs. nonsynonymous divergence 0.042), suggesting that the positive selection drove the diversification of each duplicate independently instead of maintaining their divergence. Meanwhile, codon 253 had high posterior probabilities (*P* > 0.95) of ω > 1 in Dup1 (aspartate) and Dup2 (histidine) in the branch-site model, which was consistent with the estimation of site models M2a and M8 (253D, Supplementary Table 3). Both, the branch-site and site-model tests suggested that these two duplicates were divergently selected at the specific codon with independent evolution of a high amino acid replacement rate after species divergence.

### Evidence of functional divergence of *DFR* duplicates: *in silico* analyses

Intron length of Dup1 is obviously shorter than that of Dup2, especially introns 1 and 2 (Supplementary Fig. 4). Intron length variation probably is an outcome of recombination^46^. Variable introns would increase genome diversity by permitting different recombination arrangements and would accelerate the proteome evolution by differential splicing^47^,^48^, which could benefit organism fitness and contribute not only to gene family divergence but also to species diversity and differentiation^47^. Longer introns of Dup2 incorporate significantly abundant conserved motifs identical to *cis-*acting elements (97.909 ± 7.329 vs. 116.778 ± 12.717, *P* < 0.0001, Supplementary Table 4), which implies differential regulation of gene expression. Since the accumulation of regulatory motifs reflects an evolutionary consequence of differential expression of the duplicates instead of an immediate expression in response to stimuli, we compared codon usages instead of real-time RNA expression of the duplicates to test their proposed differential regulation. Codon usage bias, indicative of the expression efficiency^49^, was suggested to stem from selection for translational efficiency^49^,^50^,^51^. Different patterns of the effective number of codons (ENCs) in Dup1 and Dup2 (Supplementary Fig. 5) supported the hypothesis of differential expression patterns inferred by intron lengths. Lower ENC (48.01 ± 0.76) and higher codon bias index (CBI, 0.32 ± 0.01) of Dup1 in comparison with Dup2 (ENC 52.31 ± 1.80, *P* = 1.426×10^−10^; CBI 0.30 ± 0.01, *P* = 3.331×10^−12^) suggested that Dup1 tends to be highly and/or rapidly expressed, with high preference for specific nucleotides in the wobble positions (optimal codons).

**Figure 3:**
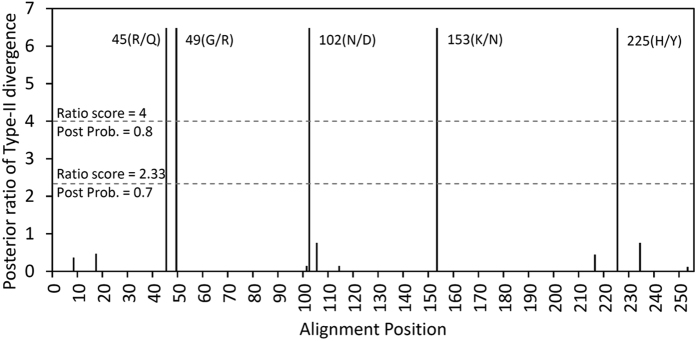
Site-specific profile for type-II functional divergence of *DFR* genes in *Scutellaria*.

We next used Gu’s statistics^52^,^53^ to test whether these two duplicated genes were functionally divergent. Gu describes two types of functional divergence, i.e., according to the evolutionary rate divergence (type-I divergence) and the change of amino acid properties (type-II divergence). The species with only one sequenced duplicate were excluded from testing with Gu’s statistics. Homogeneous evolutionary rates could not be rejected in *θ*_*I*_ (*θ*_*I*_ = 0.039 ± 0.074, *Z*-score = 0.074, *P* = 0.296), suggesting that type-I divergence was not supported, although marginal significance was detected in *θ*_*I*_ML test (*θ*_*I*_ML = 0.462 ± 0.240, LRT = 3.694, *P* = 0.055). In contrast, conserved amino acid change was rejected after 1000 bootstrap replications (*θ*_*II*_ = 1.675 ± 0.380, *Z*-score = 4.412, *P* < 0.00001), suggesting type-II functional divergence (i.e., radical change) of the *DFR* duplicates in *Scutellaria* species (Table 3). Nearly 2.25-time radical change under functional divergence than nonfunctional change (*a*_*R*_/*π*_*R*_) and 0.9% fixed radical change (*F*00,*R*) were estimated (Table 3). From the aligned DFR amino acids, 5/255 (2%) that received a ratio score > 4 (i.e., posterior probability > 0.8 or false positive < 0.2) were different between Dup1 and Dup2: 45(R/Q), 49(G/R), 102(N/D), 153(K/N), and 225(H/Y) (Fig. 3). These radical changes between DFR duplicates suggested that these duplicates have undergone a division of labor by retaining different aspects of the ancestral function to prevent redundancy, and therefore escaping the fate of nonfunctionalization.

### Evidence of functional divergence of *DFR* duplicates: differential expression in different tissues and stages

**Figure 4:**
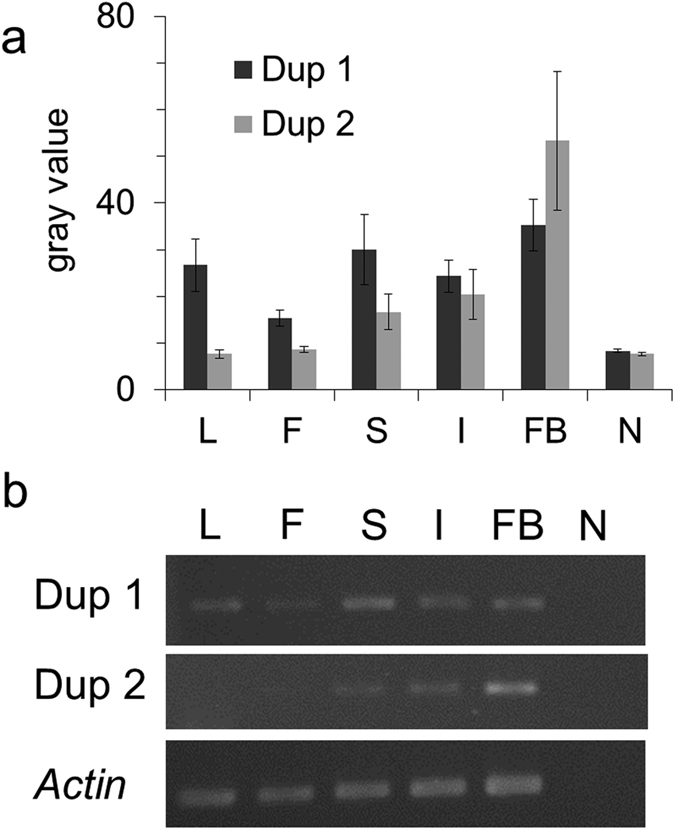
RT-PCR results of *Scutellaria playfairii DFR* Dup 1, Dup 2 and internal control (*Actin*). (**a**) The light intensity (gray value) of amplified RT-PCR products analyzed using ImageJ. The error bar represented the standard error. (**b**) The amplified RT-PCR products were visualized in the agarose gel. L: leaf; F: mature flower; S: shoot apex; I: inflorescence buds; FB: flower buds; N: No-RT negative control.

In addition to the divergent translation efficiency inferred by codon usage bias, we further compare the RNA expression between Dup 1 and Dup 2 in different tissues to validate whether these two duplicates exhibit differential expression patterns. The Dup 1 is broadly expressed in most tissues including leaves, reproductive (mature flower and flower buds) and developmental tissues (shoot apex and inflorescence buds) (Fig. 4) with slightly differential expression as reports in other model plants (e.g. accession number: AT5G42800 in TAIR, https://www.arabidopsis.org/). For example, the mature flowers revealed relatively small expression level in contrast to other tissues, while the expression of the Dup1 of the *DFR* is highest in shoot apex (Fig. 4a). In contrast, the expression of Dup 2 is restricted in organs that no expression was found in the leaf and mature flower, while it dominantly express in developmental organs, such as shoot apex, flower buds and inflorescence buds (Fig. 3a). Obviously differential expression pattern between Dup1 and Dup2 of *DFR* could be found in tissues of the leaf, mature flower, shoot apex and flower bud (Fig. 4a), but not in inflorescence buds (Fig. 4a). The expression domain of Dup 2 is therefore suggested to be limited than the ancestral gene does. Reduction of expression in one paralogs (i.e. Dup 2) implies quantitative subF between these two paralogs.

In both *in silico* analyses and RNA expression experiments, we suggested that these two paralogs of *DFR* play a role in functional subdivision at different stages and tissues in *Scutellaria*. One of the duplicates (Dup1) were expressed in all examined tissues, which may suggested to maintain ancestral functions and is only partially consistent with the definition of subF of partitioning multiple functions through complementary degeneration^54^,^55^. Such kind of functional subdivision accompanying positive selection usually attributes to the adaptation to environmental pressures and could be a solution for genetic adaptive conflict in plants^56^.

### Transient EAC explains *DFR* duplication

The EAC was suggested as an adaptive subF, in contrast to the duplication, degeneration, and complementation (DDC) model of neutral subF^8^. Due to difficulty in distinguishing EAC and DDC, several studies suggested many diagnostic features for EAC. For example, the EAC evolutionary model in *DFR* has been evidenced based on the increased nonsynonymous mutation rates and enzyme activity improvement^29^. Besides, the EAC model could also be evidenced based on adaptive change (ω > 1) in one copy with subsequently neutral subF that acts on quantitative differential expression between duplicates (Fig. 3^57^,^58^,^59^). The later feature could also be applied to predict EAC in those genes with unknown functions in descendent duplicate^60^. In the case of *Scutellaria DFR*, differential expression and radical changes in Dup1 vs. Dup2 with positive selection signals in both duplicates suggested adaptive subF, and also fit to the criteria of EAC. Under the EAC, both duplicates were expected to have a high advantageous mutation rate (ω > 1, i.e., most advantageous replacements were preserved) to overcome the mutational load and redundancy. However, due to lack of the evidence of the change of enzyme activity as well as the uncertainty of ancestral function improvement, which is usually a criterion for distinguishing EAC and DDC^61^, we cannot completely rule out the possibility of neoF or the DDC model of subF, although there is more evidence to support EAC.

**Figure 5:**
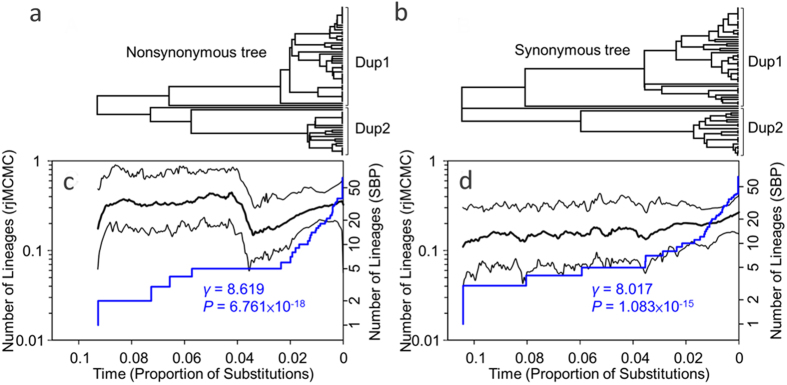
Lineage-through-time (LTT) plots inferred from trees of nonsynonymous and synonymous substitutions of *DFR* gene. (**a,b**) represent topologies of nonsynonymous and synonymous trees, respectively, with the branch lengths corresponding to the relative times (denoted as the proportion of substitutions) in figures (**c,d**). (**c,d**) represent LTT plots estimated by reversible-jump Markov chain Monte Carlo (rjMCMC) method (black lines) and a constant birth-death stochastic branching process (SBP, blue lines) based on tree topologies of (A) and (C), respectively. Significant positives of γ-statistic for the SBP-LTT plots denote the late increase of diversification rate in both trees. The x-axes indicate relative time scale since *DFR* duplication.

In Ancliff and Park’s modeling^62^, the duplicates escaping an adaptive conflict would move toward a “duplication loss of function” (DLoF) phase to decrease the long-term retention of duplicates, where one of the duplicates would evolve neutrally or at a lower evolutionary rate, and would lose its original function. Therefore, we predicted a reduction of the selection signals in one of the duplicates if this general trend would be applicable to *Scutellaria DFR.* A discovery of pseudogenes in *S. taiwanensis* Dup2 and relatively few branches with ω > 1 at the basal branching of Dup2 (Fig. 2a and Supplementary Fig. 3) implied that the gene diversification by adaptive subF was a transient, episodic evolutionary event moving toward the DLoF phase. If the hypothesis of transient adaptive subF for gene duplication were true, we expected a higher amino acid replacement rate at the beginning of gene duplication. To test this hypothesis, we compared the diversification rate dynamics in the nonsynonymous and synonymous trees. Higher diversification rate of the nonsynonymous tree at the beginning of *DFR* duplication compared with the later stage (Fig. 5c) and no obvious change of the diversification rate in the synonymous tree (Fig. 5d) verified this hypothesis. Furthermore, we found that the late burst of diversification was mostly contributed by clade Dup1 (γ = 5.690 and *P* = 1.27 × 10^−8^ vs. γ = 5.120 and *P* = 3.05 × 10^−7^ in the nonsynonymous and synonymous trees, respectively) rather than Dup2 (γ = 1.851 and *P* = 0.064 vs. γ = 2.142 and *P* = 0.032 in the nonsynonymous and synonymous trees, respectively, Table 4). The γ-statistic is a sensitive and powerful indicator detecting the change of a recent diversification rate^63^. The non-significant γ of clade Dup2 of the nonsynonymous tree implied a functional constraint or a trend of diversity loss in Dup2. The asymmetric evolutionary rates of duplicated genes and the non-varying or nearly non-varying rates of Dup2 also supported the hypothesis of transient adaptive subF moving toward the DLoF phase^62^. A selection on preexisting loci rather than diversification of new duplicates was suggested to contribute to ensuring of the normal function of the ancestors^64^, also probably explaining the asymmetric signatures of positive selection in the two duplicates (Fig. 2a and Supplementary Fig. 3).

Dosage imbalance hypothesis might comprise a possible explanation for the *DFR* DLoF phase in *Scutellaria*. Duplication of a single gene may result in a dosage imbalance in a corresponding pathway or gene network, affecting the efficiency of gene-gene interactions. Consequently, selection may favor the reversal of the duplicated genes back to a single-copy state^65^. Most species containing more than two *DFR* gene copies also possess multiple copies of other ABP genes. For example, there are two copies of *CHS* in *Zea*^66^, at least five copies of *CHS* in *Ipomoea*^67^, at least eight copies of *CHS* and one to two copies of *CHI* in *Medicago*^68^, at least 13 copies of *CHS*, four copies of *PKR*, etc. in *Lotus*^69^, at least six *CHS* and seven *CHS-*like genes, two *F3′5′H* copies, etc. in *Populus*^70^. These concerted duplication events might be related to the recent whole genome duplications in these taxa^69^,^71^,^72^,^73^. Our phylogenetic analyses (Fig. 1) are also consistent with the interpretation of recent duplication of *DFR* genes in most species. Whole genome duplication can duplicate all ABP genes at once, retaining the ideal dosage ratio. Therefore, it may prevent the dosage imbalance effect in these taxa. In contrast, *DFR* duplication in *Scutellaria* did not coincide with *CHS* duplication^31^. According to the dosage imbalance hypothesis^65^, this suggests that *Scutellaria* *DFR* should be fractionated toward single-copy state^74^ and supports our hypothesis of adaptive subF moving toward the DLoF phase.

In conclusion, we found that recombination and gene duplication episodes that followed the positive selection shaped the evolutionary scenario of *DFR*. These non-neutral mechanisms preserved the gene ancestral functions and also modified them, facilitating adaptation during species diversification. Sequence analyses and differential expressions of *DFR* duplications in *Scutellaria* basically supported the hypothesis of adaptive functional subdivision (subF) for *DFR* duplicates^29^, and further suggested that the high genetic variability accelerated by the positive selection was transient. These processes (recombination plus selection) ensure the functional diversity (pleiotropy) of this anthocyanin pathway gene^19^. Persistence of a standing genetic variation is important for the maintenance of pleiotropy^75^, explaining the decrease of diversification rate after duplication. Imbalanced positively selective pressures acting on two duplicated paralogs could decrease the risk of genetic load. The discovery of a pseudogene with lower evolutionary rates in one of the duplicated clusters suggested that the EAC evolutionary mode for subF may be difficult for long-term persistence, perhaps because of a dosage imbalance in the entire ABP pathway. Such transient process of selection of this ABP gene could have already co-influenced such pleiotropic ecological functions as pollination, UV protection, etc.

## Methods

### *Scutellaria* sampling and sequencing

Twelve *Scutellaria* species (*S. amabilis*, *S. zhongdianensis*, *S. salvifolia*, *S. altissima*, *S. lateriflora*, *S. austrotaiwanensis*, *S. indica*, *S. playfairii*, *S. tashiroi*, *S. taiwanensis*, *S. barbata*, *S. taipeiensis*) were sampled for *DFR* gene sequencing. The phylogenetically close genus *Tinnea* (*T. rhodesiana*) was used to root the *Scutellaria DFR* gene tree. All plants were grown in a greenhouse of the National Taiwan Normal University (Taipei, Taiwan) and the leaves were collected for DNA extraction. Primer pair ScDFR-F1 (5′-CACCGGCGTNTTCCAYGTTG-3′) and ScDFR-R1 (5′-GAGCAAATGTANCGNCCNTC-3′), and a forward nested primer ScDFR-F2 (5′-GGTCATCCARGTGNACNWANTG-3′) were used to amplify the *DFR* gene. The PCR products with different size length were isolated with gel extraction and cloned. At least three colonies from each gel extraction products were picked and sequenced using ABI BigDye 3.1 Terminator Cycle Sequencing Kit (Applied Biosystems, Foster City, CA, USA). All sequences were visually inspected from chromatograms from ABI PRISM^®^3730XL DNA Sequencer (Perkin-Elmer, Foster City, CA, USA). For reducing the influence of cloning error, the sequences with unique singletons were excluded for further analysis. The homology of all sequences was assessed by the bidirectional best hit (BBH) approach. Sequence alignments were conducted using the MUSCLE multiple sequence alignment software tool^76^,^77^ before further analyses.

### Data collection

To reconstruct the *DFR* gene tree and further analyze nucleotide diversity and gene recombination rate, we downloaded the *DFR* coding sequences from the NCBI GenBank using the keywords “dihydroflavonol 4-reductase” and “coding sequence”. Most entries were extracted and used except for the ones with partial or too short sequences and those of incorrectly annotated. Homology of all sequences was checked by BBH approach.

### Phylogenetic tree reconstruction

For reconstructing *DFR* gene tree of angiosperm, nucleotide sequences were translated to amino acid sequences, aligned using the MUSCLE software^76^,^77^, and the aligned amino acid sequences were further reverse translated to nucleotide sequences. Variable lengths 5′- and 3′-termini were trimmed. The aligned *DFR* genes of *Scutellaria* were divided into three data set, which are exon, intron, and total length dataset. Neighbor joining (NJ), maximum likelihood (ML), and Bayesian inference (BI) phylogenetic trees were conducted by MEGA v. 6^78^, PhyML^79^, and MrBayes v3.2^80^, respectively, to infer evolutionary relationships between homologous *DFR* genes. Maximum Composite Likelihood substitution model and pairwise deletion method were adopted to deal with the substitution and indels of alignments of NJ phylogenetic tree, while best substitution models evaluated by Bayesian Information Criterion (BIC) were adopted for ML and BI phylogenetic tree reconstruction. The best models for exon, intron, and total sequences alignments were K2P+G+I, HKY+G+I, and HKY+G+I, respectively. One million MCMC steps with four chains were sampled in the BI analysis. A 1000-times bootstrap replication, aLRT, and posterior probability were set for evaluating the supporting values of lineage grouping for the NJ, ML, and BI trees, respectively.

### Recombination

The recombination rate of *DFR* gene was estimated using Hudson’s estimator *R*^81^. Hudson’s *R* was then divided by an average nucleotide distance to obtain the recombination rate between the adjacent sites. We also estimated the minimum number of recombination events (*Rm*) using a four-gamete test, a method for detecting historical recombination events^82^. The *ZZ* statistic was used for detecting intragenic recombination^83^. Since the *ZZ* statistic calculates the differences in linkage disequilibrium between the overall pairwise site comparison and the adjacent sites, it is more sensitive to the increase of recombination and less affected by parallel mutation^83^. Coalescent simulations of *Rm* and *ZZ* were performed for genera with sequence number > 9 (i.e., *Aegilops*, *Allium*, *Brassica*, *Fragaria*, *Iochroma*, *Ipomoea*, *Nicotiana*, *Prunus*, *Pyrus*, *Scutellaria*, *Solanum*, and *Triticum*). All recombination analyses were conducted by DnaSP 5.10^84^.

### Tajima’s *D* statistic

Tajima’s *D* analysis was used for testing the difference in nucleotide diversity estimated by a pairwise nucleotide difference (π) and an index of diversity estimated by the numbers of segregating sites (θ_W_). Tajima’s *D* was usually used as a population-level neutrality test, while we used this statistic to evaluate the disparity of ancestral nucleotide variation and newly derived polymorphisms, where the former led to a larger amount of common polymorphisms and the latter resulted in abundant rare alleles.

### Conserved motifs in introns

Conserved motifs in *Scutellaria DFR* introns were identified by searching the database of plant *cis*-acting regulatory DNA elements, NEWPLACE^85^. The number of these putative *cis*-acting elements was counted.

### Codon usages

The ENCs and CBI of each duplicate *Scutellaria DFR* gene were estimated by DnaSP 5.10^84^. Significant differences of ENCs and CBI between paralogs were calculated by Student’s *t*-test. ENC plot was generated to evaluate the degrees of deviation from the neutral expectation in the absence of selection.

### Topological test

*Scutellaria* samples were used to investigate the evolutionary scenario of duplication events. We first tested two evolutionary hypotheses: (1) speciation after duplication and (2) duplication after speciation (Supplementary Fig. 1). We used the baseml program in PAML v.4.2^86^ to produce the log-likelihoods of site-patterns of both trees and performed the AU, KH, and SH tests to evaluate the best tree by CONSEL^87^.

### Lineage-through-time analysis

To compare the change of nonsynonymous and synonymous diversification rates of *DFR*, NJ trees were reconstructed considering sites of nonsynonymous substitutions only or synonymous substitutions only. The substitution model was set up as the Nei-Gojobori method (Proportion). The reconstructed nonsynonymous and synonymous NJ trees were used as the input trees to reconstruct the lineage-through-time (LTT) plots using the constant birth-death stochastic branching process (SBP)^88^ and reversible-jump Markov chain Monte Carlo (rjMCMC) methods^89^. Genealogical time frame was scaled using the proportion of substitutions. The γ-statistic was used to evaluate the variation pattern of diversification rate through time^90^. The LTT analyses were implemented in R.

### Estimating evolutionary rates of lineages and codons

For *Scutellaria* samples, both nonsynonymous (dN) and synonymous substitution rates (dS) and dN/dS ratio (ω) of every lineage were estimated using the branch model (free-ratio) analysis with codeml program in PAML v.4.2^86^. We used both intron tree and exon tree as the input user trees because neither tree can be reciprocally rejected by the topological test (Supplementary Table 1). Constant model (M0) was used as the null model in comparisons by likelihood-ratio test (LRT) using the Chi-square distribution to assess significance. Branch-site model A was used to test whether each duplicate cluster had a relatively high divergent rate (i.e., foreground branch) under the condition of constraint evolutionary rate of another duplicate cluster (i.e., background branch). Both modes of “lineage of whole clade of each duplicate” (marked as #1) and “all lineages of each duplicate clade” (marked as $1) were set as foregrounds for testing the persistence of positive selection. Fixed ω = 1 of the foreground branch was used as a null model in comparison by LRT. The site model M1a (nearly neutral model) vs. M2a (positive selection model), M7 (beta) vs. M8 (beta&ω), and M8a (beta&ω_s_ = 1) vs. M8 were compared to identify a positively selected codon.

### Functional divergence

The function divergence between duplicates of *Scutellaria DFR* gene was inferred by type-I (Gu99) and type-II divergence analyses. Type-I and type-II divergence suggests heterogeneous evolutionary rates and radical changes to biochemical properties (charge positive/negative, hydrophilic/hydrophobic) between the duplicates, respectively. Divergence indices of both type-I and type-II were calculated with 500 bootstrap replications using DIVERGE version 3^91^. Posterior ratio was used to calculate the posterior probability of sites with type-II divergent functions^92^.

### Expression analysis

To validate expressional differences between duplicates of *Scutellaria DFR* genes, expression patterns of duplicates among different tissues were evaluated using reverse-transcriptional PCR (RT-PCR). Five tissues (leaf, mature flower, shoot apex, inflorescence buds, and flower buds) from *S. playfairii* were selected for expression level examination. Total RNA of these tissues were extracted using TRIzol reagent (Ambion, Thermo Fisher Scientific Inc., USA), and 1 *μ*g RNA was reverse-transcribed using ProtoScript II First Strand cDNA Synthesis Kit (New England Biolabs, USA). Specific primers for two *DFR* paralogs were designed for amplification: cDFR-1F (5′-TGTTGAACAACACCAAAAACCAG-3′) and cDFR-1R (5′-GTGGTGCGCTTCATTCCCAG-3′ for Dup 1; cDFR-2F (5′-CGTTGAAGAACACCAAAAACCAC) and cDFR-2R (5′-GTAGTGGGCTTCATTCCCAG-3′) for Dup 2. The *actin* gene was adopted as internal control (designed forward primer: 5′-AGCAACTGGGATGATATGGA-3′; reverse primer: 5′-CCATCACCAGAGTCGAGAAC-3′). Three cycles (27, 29, 31 cycles) on the thermocycler were conducted to ensure the amplicons not over-saturated in PCR. Finally, the 29 cycles were adopted for expression analysis. Three biological repeats were conducted, and light intensity of each paralogs products was measured and compared using the ROI manager implemented in ImageJ^93^.

## Additional Information

**How to cite this article**: Huang, B.-H. *et al*. Imbalanced positive selection maintains the functional divergence of duplicated *DIHYDROKAEMPFEROL 4-REDUCTASE* genes. *Sci. Rep.* **6**, 39031; doi: 10.1038/srep39031 (2016).

**Publisher's note:** Springer Nature remains neutral with regard to jurisdictional claims in published maps and institutional affiliations.

## Supplementary Material

Supplementary Information

## Figures and Tables

**Table 1:** Recombination analyses estimated by *R*, *Rm*, and *ZZ* statistic. Both observed and coalescent simulations are shown.

Taxa	Tajima’s *D*	*P*	*R* (per gene)^a^	*R* (between adjacent site)^a^	*Rm*^a^	Average *Rm*^b^	95% CI, lower limit^b^	95% CI, upper limit^b^	*P*^b^,^c^	*ZZ*^a^	Average *ZZ*^b^	95% CI, lower limit^b^	95% CI, upper limit^b^	*P*^b^,^d^
Angiosperm	3.74047	<0.001	101	0.1322	37	—	—	—	—	0.0334	—	—	—	—
*Scutellaria*	−0.64289	>0.1	2.9	0.0038	25	80.805	64	99	1	0.1477	0.0006	−0.0375	0.0378	0
*Ipomoea*	−0.51051	>0.1	0.001	0	24	100.601	83	121	1	0.0492	−0.0005	−0.0367	0.0407	0.013
*Aegilops*	−0.1985	>0.1	41.3	0.0538	8	28.942	19	39	1	0.0265	−0.0006	−0.0734	0.0779	0.224
*Triticum*	−0.85068	>0.1	14.1	0.0183	8	34.079	24	45	1	0.1629	0.0015	−0.0576	0.073	0.001
*Allium*	0.13892	>0.1	89.3	0.1168	1	2.297	0	6	0.658	0.1731	0.0056	−0.1829	0.2098	0.057
*Brassica*	0.68813	>0.1	16.7	0.0219	1	7.581	3	13	0.999	0.0279	−0.0028	−0.1357	0.1497	0.27
*Fragaria*	2.10125	<0.05	0.001	0	5	112.14	93	133	1	0.0236	−0.0003	−0.0377	0.0369	0.1
*Iochroma*	−1.19213	>0.1	41.2	0.0541	0	11.124	5	18	1	0.0464	−0.0003	−0.1086	0.1172	0.195
*Nicotiana*	0.59302	>0.1	12.2	0.016	1	25.493	17	35	1	0.0308	0	−0.0817	0.0773	0.21
*Prunus*	1.23996	>0.1	4	0.0052	1	14.923	8	23	1	0.0673	−0.0002	−0.1016	0.1071	0.089
*Pyrus*	−0.53469	>0.1	45.1	0.059	1	6.887	2	13	0.995	−0.0177	−0.002	−0.1402	0.1532	0.577
*Solanum*	−0.03248	>0.1	30	0.0393	1	14.161	8	22	1	−0.0185	−0.0002	−0.1047	0.1266	0.631

^a^Observed value.

^b^Coalescent simulation.

^c^Probability of obtaining values of the *Rm* statistic equal to or greater than the observed value; the probabilities were obtained from coalescent simulations with free recombination.

^d^Probability of obtaining values of the *ZZ* statistic equal to or greater than the observed value; the probabilities were obtained from coalescent simulations with no recombination.

**Table 2:** Results of branch-site model analysis and the likelihood ratio test for the foreground branches of *Scutellaria DFR* duplicates.

	Foreground branch	np	lnL	2ΔL	*P*	Proportion	Background ω	Foreground ω	PS site (Pr ω > 1)
#1	Dup1 fixed ω = 1	136	−2629.741	0.4763	0.4556	p0 = 0	ω0 = 0.0355	ω0 = 0.0355	None
						p1 = 0	ω1 = 1	ω1 = 1	
						p2a = 0.8073	ω2a = 0.0355	ω2a = 1	
						p2b = 0.1927	ω2b = 1	ω2b = 1	
#1	Dup1	137	−2629.503			p0 = 0	ω0 = 0.0358	ω0 = 0.0358	
						p1 = 0	ω1 = 1	ω1 = 1	
						p2a = 0.8081	ω2a = 0.0358	ω2a = 64.0267	
						p2b = 0.1919	ω2b = 1	ω2b = 64.0267	
#1	Dup2 fixed ω = 1	136	−2629.61	−0.2294	#NUM!	p0 = 0.0165	ω0 = 0.0356	ω0 = 0.0356	None
						p1 = 0.0039	ω1 = 1	ω1 = 1	
						p2a = 0.7907	ω2a = 0.0356	ω2a = 1	
						p2b = 0.1889	ω2b = 1	ω2b = 1	
#1	Dup2	137	−2629.725			p0 = 0	ω0 = 0.0359	ω0 = 0.0359	
						p1 = 0	ω1 = 1	ω1 = 1	
						p2a = 0.8079	ω2a = 0.0359	ω2a = 48.6756	
						p2b = 0.1921	ω2b = 1	ω2b = 48.6756	
$1	Dup1 fixed ω = 1	136	−2628.349	18.0661	1.12E–05	p0 = 0.7837	ω0 = 0.0337	ω0 = 0.0337	
						p1 = 0.1114	ω1 = 1	ω1 = 1	
						p2a = 0.0919	ω2a = 0.0337	ω2a = 1	
						p2b = 0.0131	ω2b = 1	ω2b = 1	
$1	Dup1	137	−2619.314			p0 = 0.7921	ω0 = 0.0412	ω0 = 0.0412	253D (1.000)
						p1 = 0.1929	ω1 = 1	ω1 = 1	
						p2a = 0.0121	ω2a = 0.0412	ω2a = 6.4153	
						p2b = 0.0029	ω2b = 1	ω2b = 6.4153	
$1	Dup2 fixed ω = 1	136	−2629.786	8.845	0.0016	p0 = 0.7754	ω0 = 0.0327	ω0 = 0.0327	
						p1 = 0.1800	ω1 = 1	ω1 = 1	
						p2a = 0.0362	ω2a = 0.0327	ω2a = 1	
						p2b = 0.0084	ω2b = 1	ω2b = 1	
$1	Dup2	137	−2625.364			p0 = 0.7966	ω0 = 0.0396	ω0 = 0.0396	253 H (0.978)
						p1 = 0.1954	ω1 = 1	ω1 = 1	
						p2a = 0.0064	ω2a = 0.0396	ω2a = 11.6666	
						p2b = 0.0016	ω2b = 1	ω2b = 11.6666	

Designations #1 and $1 indicate that the foreground branch is the lineage of entire clades or all lineages of the clade, respectively.

**Table 3:** Type-I and type-II functional divergence estimated by Gu’s statistics.

Type-I Divergence		Type-II Divergence	
*θ*_*I*_ ± SE^a^	0.039 ± 0.074	*θ*_*II*_ ± SE^d^	1.675 ± 0.380
*Z*-score (*P* value)	0.074 (0.296)	*Z*-score (*P* value)	4.412 (<0.00001)
*θ*_*I*_ML ± SE^b^	0.462 ± 0.240	*a*_*R*_/*π*_*R*_^e^	4.453
LRT *θ*_*I*_ (*P* value)^c^	3.694 (0.055)	*G*_*R*_/*G*_*C*_^f^	1.456
		*N*/*C*/*R*^g^	230.9/8.3/14.8
		*F*00,*N*/*C*/*R*^h^	0.810/0.021/0.009

^a^The estimate of functional divergence coefficient *θ*_*I*_ with standard error by a model-free method.

^b^Maximum likelihood estimate of *θ*_*I*_ and standard error.

^c^The log score for the likelihood ratio test against the null *θ*_*I*_ = 0.

^d^The estimate of functional divergence coefficient *θ*_*II*_ with standard error.

^e^The ratio of radical change under functional divergence (*a*_*R*_) versus nonfunctional divergence (*π*_*R*_).

^f^The ratio of proportion of radical change (*G*_*R*_) versus conserved change (*G*_*C*_);

^g^The numbers of sites indicate no difference (*N*), conserved difference (*C*), and radical differences (*R*).

^h^Proportion of no change (*F*00,*N*), radical change (*F*00,*R*), and conserved change (*F*00,*C*) of amino acids between clusters but “no change” within clusters.

**Table 4:** γ-statistic of nonsynonymous and synonymous trees of *DFR* in *Scutellaria*.

	γ	P
Nonsyn	8.619	6.76E–18
Nonsyn Dup1	5.690	1.27E–08
Nonsyn Dup2	1.851	0.064
Synonym	8.017	1.08E–15
Synonym Dup1	5.120	3.05E–07
Synonym Dup2	2.142	0.032

Nonsyn: nonsynonymous tree of *DFR.*

Synonym: synonymous tree of *DFR.*
