# Supplementary material for: Imbalanced positive selection maintains the functional divergence of duplicated DIHYDROKAEMPFEROL 4-REDUCTASE genes
Source: Sci Rep. 2016 Dec 14;6:39031. doi: 10.1038/srep39031 (PMC5155217; doi:10.1038/srep39031)
Supplement: Supplementary Information [file srep39031-s1.pdf]

Supplementary information for "Imbalanced positive selection maintains the functional divergence of duplicated *DIHYDROKAEMPFEROL 4-REDUCTASE* genes"

Yi-Wen Chen[1,]†, Bing-Hong Huang[2,]†, Chia-Lung Huang[2], Jian Gao[3], Pei-Chun Liao[2,]*

Supplementary Table 1 Topological tests to determine the best input tree for PAML analyses. The result revealed that the exon tree could not reject better tree topology reconstructed by intron sequences.

Rank	Item	obs	au	np	bp	pp	kh	sh	wkh	wsh
1	intron tree	-49.4	0.797	0.845	0.842	1.000	0.849	0.849	0.849	0.849
2	extron tree	49.4	0.203	0.155	0.158	4.00E-22	0.151	0.151	0.151	0.151

au, *P*-value of the approximately unbiased test.
np, bootstrap probability of the selection.
bp, same as np but calculated directly from a set of replicates with r_k=1, where r_k is the scale for K sets of replicates.
pp, Bayesian posterior probability (PP) calculated by the BIC approximation.
kh, *P*-value of the Kishino-Hasegawa (KH) test.
sh, *P*-value of the Shimodaira-Hasegawa (SH) test.
wkh, *P*-value of the weighted Kishino-Hasegawa (WKH) test.
wsh, *P*-value of the weighted Shimodaira-Hasegawa (WSH) test.

Supplementary Table 2 Topological tests for two evolutionary hypotheses of *Scutellaria DFR*. The result showed that the hypothesis “speciation after duplication” was the best scenario, and the hypothesis “duplication after speciation” was rejected by all tests (P<0.05).

Rank	Item (Hypothesis)	obs	au	np	bp	pp	kh	sh	wkh	wsh
1	H1: Speciation after duplication	-436.7	0.994	0.995	0.996	1.000	0.993	0.993	0.993	0.993
2	H2: Duplication after speciation	436.7	0.006	0.005	0.004	2E-190	0.007	0.007	0.007	0.007

au, *P*-value of the approximately unbiased test.
np, bootstrap probability of the selection.
bp, same as np, but calculated directly from a set of replicates with r_k=1, where r_k is the scale for K sets of replicates.
pp, Bayesian posterior probability (PP) calculated by the BIC approximation.
kh, *P*-value of the Kishino-Hasegawa (KH) test.
sh, *P*-value of the Shimodaira-Hasegawa (SH) test.
wkh, *P*-value of the weighted Kishino-Hasegawa (WKH) test.
wsh, *P*-value of the weighted Shimodaira-Hasegawa (WSH) test.

Supplementary Table 3 Summary of codon-based model analyses of PAML for *DFR* of *Scutellaria* species.

Model	np	*L*	ω	Parameters	2Δ*L*	*P*
M0	136	-3008.326	0.1994	ω=0.19940	107.168	0.007
Free-ratio	269	-2954.742				
M1a	137	-2960.644	0.2156	ω0 = 0.08135 (p0 = 0.85384)<br>ω1 = 1.000 (p1 = 0.14616)	25.101	1.77E-06
M2a	139	-2948.094	0.2642	ω0 = 0.08323 (p0 = 0.84611)<br>ω1 = 1.000 (p1 = 0.14990)<br>ω2 = 11.00179 (p2 = 0.00398)		
M7	137	-2963.044	0.2183	α = 0.21788, β = 0.77395	28.835	2.74E-07
M8a	138	-2960.352	0.2009	α = 0.61415, β = 4.81459<br>p0 = 0.89683<br>p1 = 0.10317; ω1 = 1.000	23.451	3.33E-07
M8	139	-2948.626	0.2408	α = 0.27724, β = 1.10158<br>p0 = 0.99600<br>p1 = 0.00400; ω1 = 10.85527		

L, Log-likelihood of the data; ω, Mean dN⁄dS ratio for the entire gene.
Both M2a and M8 suggest that the codon at site 253D (considering gaps) was positively selected [*Pr*(ω>1) = 1.000] using Bayes Empirical Bayes (BEB) analysis.

Supplementary Table 4 Inferred conserved motifs identical to the *cis*-acting elements in *DFR* introns in *Scutellaria*. The average numbers and standard deviations of the putative *cis*-acting elements are shown.

Cis-acting elements	Motif	Dup1		Dup2	
		Average	Std	Average	Std
-10PEHVPSBD	TATTCT	0.545	0.498	1.000	0.500
2SSEEDPROTBANAPA	CAAACAC	1.000	0.426	1.000	0.500
AACACOREOSGLUB1	AACAAAC	0.000	0.000	1.000	0.707
ABRELATERD1	ACGTG	1.091	0.514	0.125	0.331
ABRERATCAL	MACGYGB	1.091	0.514	0.000	0.000
ACGTABOX	TACGTA	0.000	0.000	0.750	0.968
ACGTATERD1	ACGT	3.091	1.781	2.500	1.323
AMYBOX1	TAACARA	0.000	0.000	0.375	0.484
AMYBOX2	TATCCAT	0.818	0.386	0.000	0.000
ANAERO1CONSENSUS	AAACAAA	0.000	0.000	0.125	0.331
ARR1AT	NGATT	4.727	1.135	7.750	1.299
ASF1MOTIFCAMV	TGACG	0.000	0.000	0.375	0.484
BIHD1OS	TGTCA	0.091	0.287	1.750	0.661
BOXIINTPATPB	ATAGAA	0.818	0.386	0.625	0.484
BOXLCOREDCPAL	ACCWWCC	0.091	0.287	0.000	0.000
CAATBOX1	CAAT	2.818	0.386	7.500	1.581
CACTFTPPCA1	YACT	8.364	0.771	12.750	0.968
CANBNNAPA	CNAACAC	1.091	0.668	1.000	0.500
CAREOSREP1	CAACTC	1.727	0.445	0.625	0.696
CARGCW8GAT	CW_8G	0.000	0.000	0.500	0.866
CATATGGMSAUR	CATATG	1.818	0.575	1.500	0.866
CBFHV	RYCGAC	1.000	0.000	1.500	0.500
CCAATBOX1	CCAAT	0.000	0.000	0.250	0.433
CIACADIANLELHC	$CAAN_4ATC$	1.091	0.287	1.000	0.500
CPBCSPOR	TATTAG	1.000	0.000	1.125	0.599
CTRMCAMV35S	TCTCTCTCT	0.364	0.481	0.000	0.000
CURECORECR	GTAC	2.000	0.853	1.750	1.854
DOFCOREZM	AAAG	3.000	0.000	4.250	1.479
DPBFCOREDCDC3	ACACNNG	0.727	0.617	1.750	0.433
EBOXBNNAPA	CANNTG	5.273	1.763	7.000	2.236
EECCRCAH1	GANTTNC	4.000	1.128	4.500	0.500
ELRECOREPCRP1	TTGACC	0.000	0.000	0.125	0.331
GAREAT	TAACAAR	0.000	0.000	0.375	0.484
GATABOX	GATA	5.000	1.128	5.625	0.696
GT1CONSENSUS	GRWAAW	5.273	0.750	6.250	1.090
GT1GMSCAM4	GAAAAA	0.909	0.287	1.750	0.433
GT1MOTIFPSRBCS	KWGTGRWAAWRW	0.455	0.498	0.625	0.484
GTGANTG10	GTGA	2.727	0.750	3.875	0.331
IBOX	GATAAG	0.909	0.287	0.125	0.331
IBOXCORE	GATAA	2.000	0.739	0.250	0.433
IBOXCORENT	GATAAGR	0.909	0.287	0.125	0.331
INTRONUPPER	MAGGTAAGT	0.000	0.000	0.375	0.484
MARABOX1	AATAAAYAAA	0.000	0.000	0.125	0.331
MARTBOX	TTWTWTTWTT	0.182	0.575	0.875	0.331
MYB1AT	WAACCA	0.182	0.386	0.375	0.484
MYB2AT	TAACTG	0.000	0.000	0.250	0.433
MYB2CONSENSUSAT	YAACKG	0.364	0.481	0.250	0.433
MYBCORE	CNGTTR	0.455	0.498	1.125	0.599
MYBCOREATCYCB1	AACGG	0.364	0.481	0.000	0.000
MYBGAHV	TAACAAA	0.000	0.000	0.375	0.484
MYBPLANT	MACCWAMC	0.182	0.386	0.000	0.000
MYBPZM	CCWACC	0.091	0.287	0.000	0.000
MYBST1	GGATA	1.000	0.603	0.125	0.331
MYCATERD1	CATGTG	0.182	0.386	1.000	0.866
MYCATRD22	CACATG	0.182	0.386	1.000	0.866

Cis-acting elements	Motif	Dup1		Dup2	
		Average	Std	Average	Std
MYCCONSENSUSAT	CANNTG	5.273	1.763	7.000	2.236
NAPINMOTIFBN	TACACAT	0.182	0.386	0.000	0.000
NODCON1GM	AAAGAT	0.000	0.000	0.125	0.331
NODCON2GM	CTCTT	0.364	0.481	1.000	0.500
NTBBF1ARROLB	ACTTTA	0.545	0.498	0.750	0.433
OSE1ROOTNODULE	AAAGAT	0.000	0.000	0.125	0.331
OSE2ROOTNODULE	CTCTT	0.364	0.481	1.000	0.500
POLASIG1	AATAAA	0.182	0.575	1.000	0.500
POLASIG3	AATAAT	0.091	0.287	1.375	0.484
POLLEN1LELAT52	AGAAA	5.818	0.386	5.500	1.581
PREATPRODH	ACTCAT	0.091	0.287	0.375	0.484
PRECONSCRHSP70A	$SCGAYNRN_{15}HD$	0.091	0.287	0.875	0.331
QARBNEXTA	AACGTGT	0.909	0.287	0.000	0.000
RAV1AAT	CAACA	1.909	0.514	3.375	0.992
RAV1BAT	CACCTG	0.000	0.000	0.125	0.331
RBCSCONSENSUS	AATCCAA	0.727	0.445	0.000	0.000
REALPHALGLHCB21	AACCAA	0.909	0.287	0.375	0.484
ROOTMOTIFTAPOX1	ATATT	2.636	0.481	3.500	1.500
S1FBOXSORPS1L21	ATGGTA	0.000	0.000	0.125	0.331
SEBFCONSSTPR10A	YTGTCWC	0.000	0.000	0.875	0.331
SEF1MOTIF	ATATTTAWW	0.000	0.000	0.625	0.484
SEF3MOTIFGM	AACCCA	0.091	0.287	0.000	0.000
SEF4MOTIFGM7S	RTTTTTR	1.182	0.575	0.750	0.433
SITEIIATCYTC	TGGGCY	0.182	0.386	0.000	0.000
SORLIP1AT	GCCAC	0.000	0.000	0.125	0.331
SORLIP5AT	GAGTGAG	0.000	0.000	0.625	0.484
SP8BFIBSP8BIB	TACTATT	0.909	0.287	0.000	0.000
SREATMSD	TTATCC	0.182	0.386	0.000	0.000
SURE2STPAT21	AATACTAAT	0.000	0.000	0.250	0.433
SV40COREENHAN	GTGGWWHG	0.182	0.386	0.000	0.000
T/GBOXATPIN2	AACGTG	1.091	0.514	0.000	0.000
TAAAGSTKST1	TAAAG	1.000	0.426	1.125	0.781
TATABOX2	TATAAAT	0.000	0.000	0.750	0.433
TATABOX3	TATTAAT	0.091	0.287	0.125	0.331
TATABOX4	TATATAA	0.182	0.386	0.000	0.000
TATABOX5	TTATTT	0.182	0.575	1.250	0.829
TATCCACHVAL21	TATCCAC	0.000	0.000	0.125	0.331
TATCCAOSAMY	TATCCA	1.000	0.603	0.125	0.331
TATCCAYMOTIFOSRAMY3D	TATCCAY	0.818	0.386	0.125	0.331
TGTCACACMCUCUMISIN	TGTCACA	0.000	0.000	0.875	0.331
WBBOXPCWRKY1	TTTGACY	0.000	0.000	0.125	0.331
WBOXATNPR1	TTGAC	0.000	0.000	0.625	0.696
WBOXHVISO1	TGACT	0.000	0.000	1.000	0.500
WBOXNTERF3	TGACY	0.818	0.386	1.875	0.599
WRKY71OS	TGAC	0.909	0.514	4.000	0.866

Supplementary Fig. 1 Phylogenetic analysis of angiosperm *DFR*.

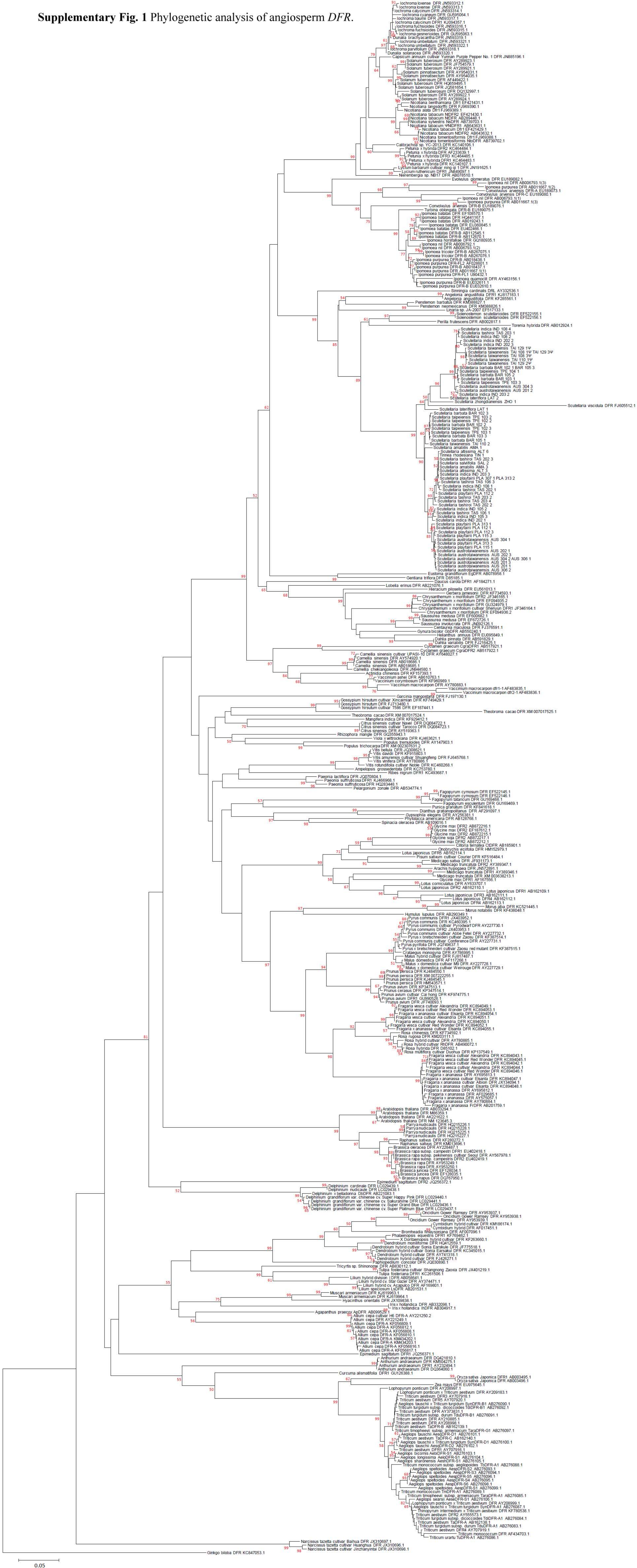

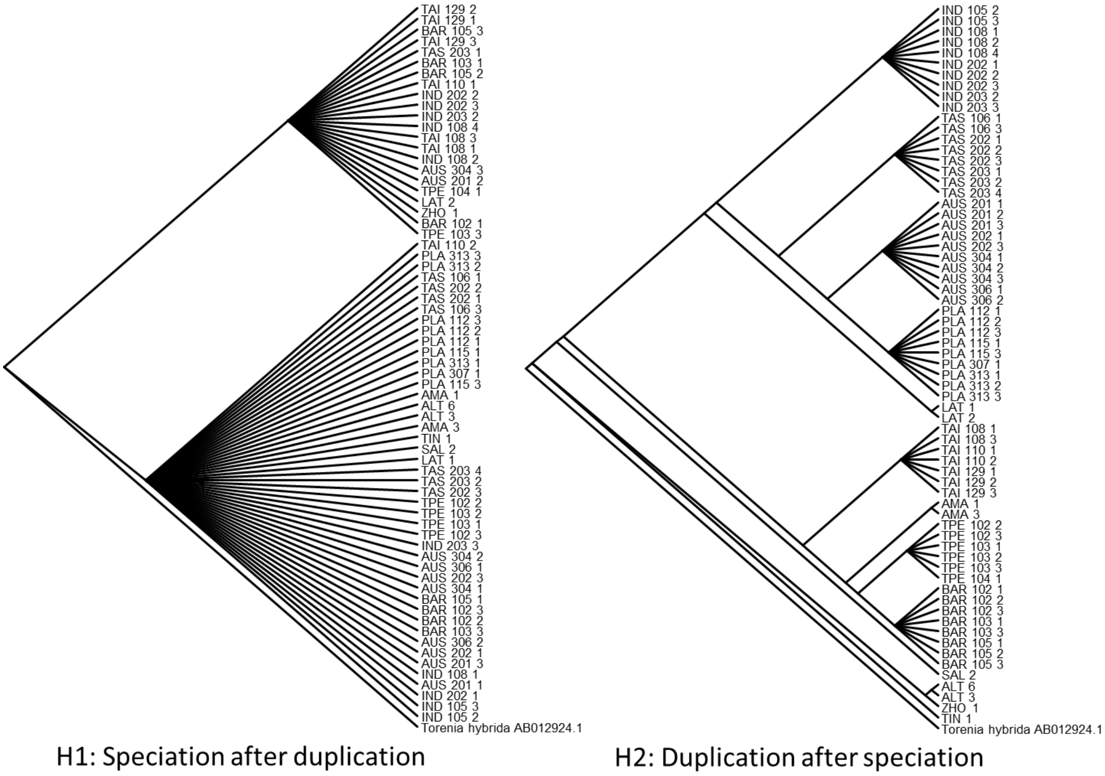

Supplementary Fig. 2 Tree topologies of duplication hypotheses of *Scutellaria DFR*.

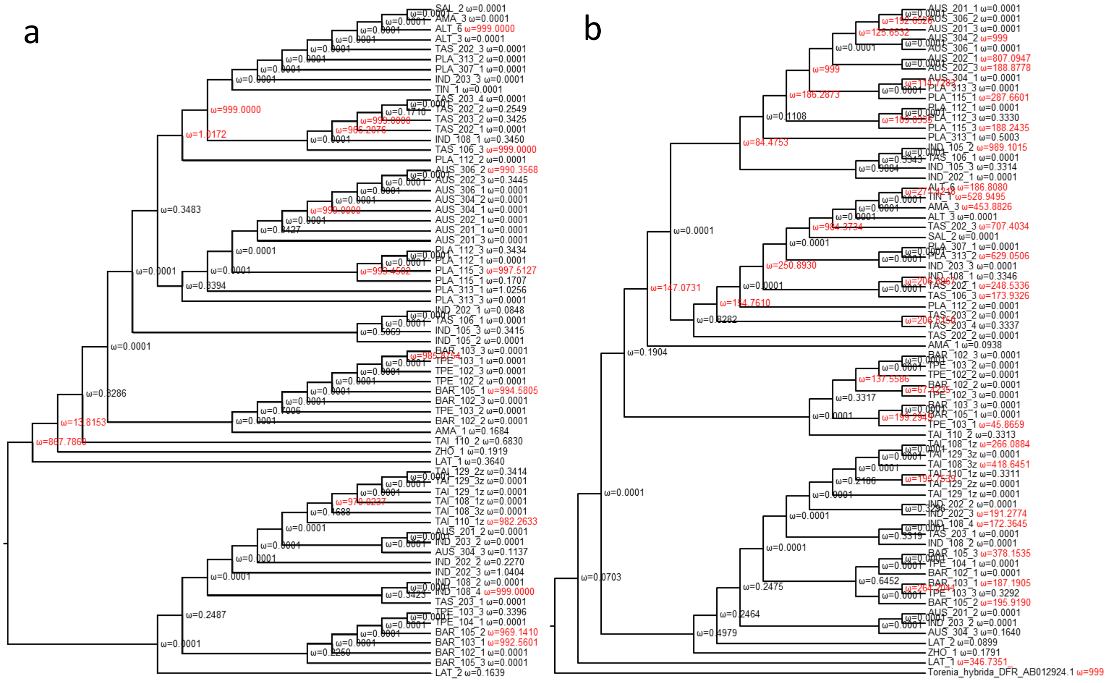

Supplementary Fig. 3 The ω values of each branch inferred by the free-ratio model of PAML using (a) intron tree and (b) exon tree as the input user tree.

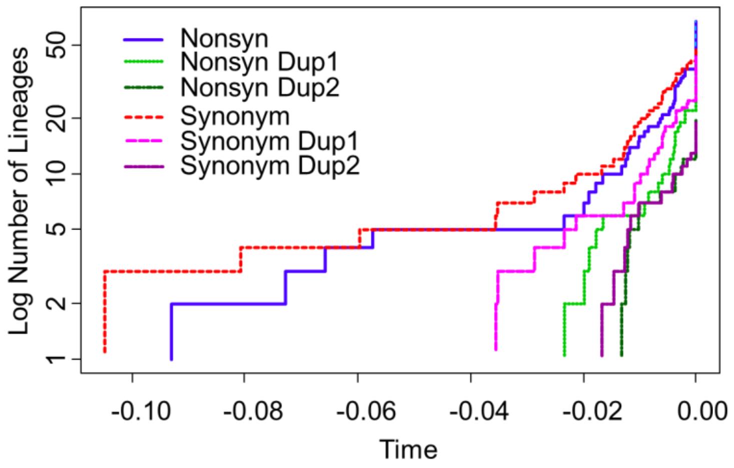

Supplementary Fig. 4 Lineage-through-time (LTT) plots of nonsynonymous and synonymous trees of *Scutellaria DFR*.

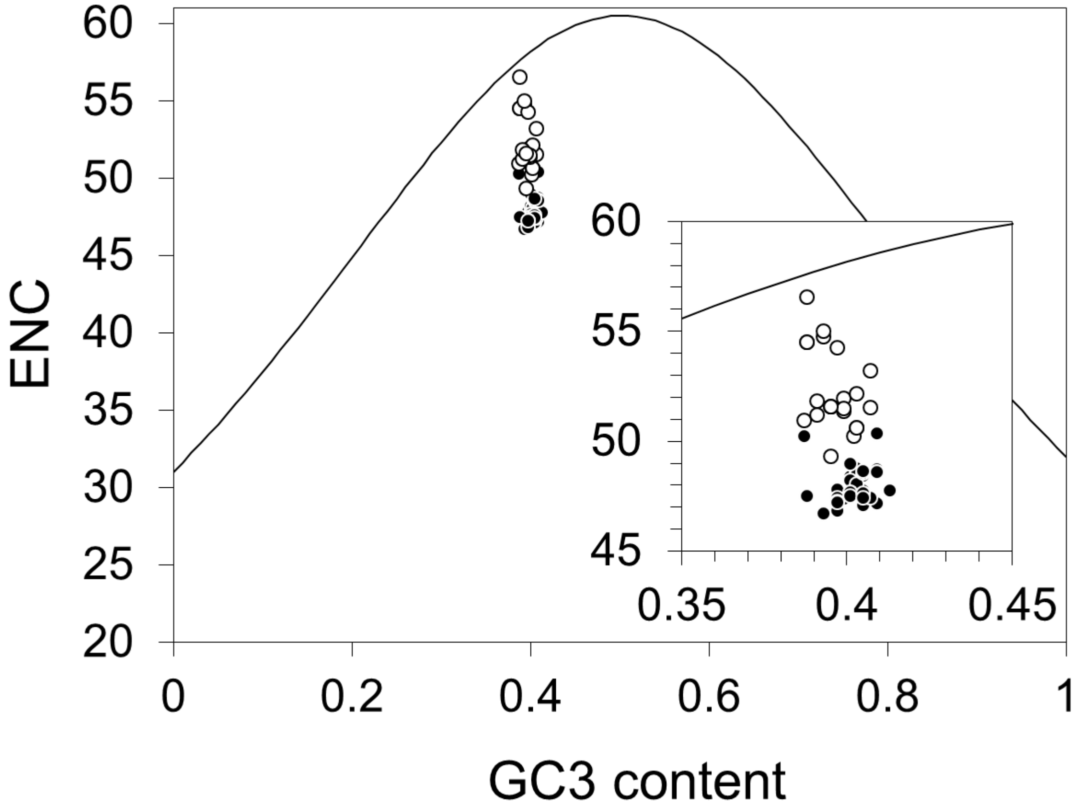

Supplementary Fig. 5 ENC-plot depicting the effective number of codons (ENCs) plotted against G+C content at synonymous third codon positions (GC3) of Dup1 (filled circles) and Dup2 (open circles) of *Scutellaria DFR*. The curve denotes the relationship between GC3 and ENC in the absence of selection (neutral). The inner panel zooms in on the ENC plot of *Scutellaria DFR*.
